# Testing in Football: A Narrative Review

**DOI:** 10.3390/sports12110307

**Published:** 2024-11-13

**Authors:** Elliott C. R. Hall, George John, Ildus I. Ahmetov

**Affiliations:** 1 Faculty of Health Sciences and Sport, University of Stirling, Stirling FK9 4UA, UK; 2 School of Sport and Exercise Sciences, Liverpool John Moores University, Liverpool L3 3AF, UK; 3 Transform Specialist Medical Centre, Dubai 119190, United Arab Emirates; drjohnsportsfit@hotmail.com; 4 Laboratory of Genetics of Aging and Longevity, Kazan State Medical University, 420012 Kazan, Russia; 5 Sports Genetics Laboratory, St. Petersburg Research Institute of Physical Culture, 191040 St. Petersburg, Russia

**Keywords:** testing, monitoring, soccer, screening, talent ID

## Abstract

Football clubs regularly test and monitor players, with different approaches reflecting player age and competitive level. This narrative review aims to summarise justifications for testing and commonly used testing protocols. We also aim to discuss the validity and reliability of specific tests used to assess football players and provide a holistic overview of protocols currently used in football or those demonstrating potential utility. The PubMed, SportDiscus, and Google Scholar databases were screened for relevant articles from inception to September 2024. Articles that met our inclusion criteria documented tests for several purposes, including talent identification or the assessment of growth/maturation, physiological capacity, sport-specific skill, health status, monitoring fatigue/recovery, training adaptation, and injury risk factors. We provide information on specific tests of anthropometry, physical capacity, biochemical markers, psychological indices, injury risk screening, sport-specific skills, and genetic profile and highlight where certain tests may require further evidence to support their use. The available evidence suggests that test selection and implementation are influenced by financial resources, coach perceptions, and playing schedules. The ability to conduct field-based testing at low cost and to test multiple players simultaneously appear to be key drivers of test development and implementation among practitioners working in elite football environments.

## 1. Introduction

Football is among the world’s most popular sports in terms of participation and spectatorship, with considerable financial incentives related to professional status. This contributes to the investment made by professional clubs in identifying and developing talented players from an early age to receive specialist coaching and training [1]. Traditionally, the selection of the most talented young footballers relied on the subjective judgement of expert coaches and/or scouts [1]. However, a range of objective measures are now available to support selection decisions and monitor the progress of individual players advancing toward adulthood. Similarly, professional football clubs routinely conduct a spectrum of tests for a multitude of purposes, including monitoring health status, screening for injury risk, assessing physiological capacity, quantifying training adaptation, and evaluating sport-specific skill performance. The choice, regularity, and analysis of tests vary according to competitive level and the financial budget available for specialist staff and/or testing resources and should be balanced against the time required to test players within busy competitive schedules. The purpose of this review is to summarise the variety of testing categories and tests employed in competitive football and, secondly, to highlight where certain testing procedures may have greater utility at different ages and/or levels of competition. Specifically, we discuss tests used to assess anthropometry, physiological capacity, sport-specific performance, biomarkers, psychology, injury risk, and genetic profile, including the rationale and/or evidence for using these tests in football.

## 2. Literature Search

Research studies and review articles were identified using a literature search of the PubMed, SportDiscus, and Google Scholar databases from inception to September 2024. Article titles, abstracts, and keywords were screened for key terms using the conjunctions “OR” and “AND” with truncation “*”. Combinations of Boolean phrases were comprised of various search terms, including (but not limited to) ‘Soccer’, ‘Football’, ‘Testing, ‘Tests’, ‘Assessment’, ‘Monitoring’, ‘Screening’, ‘Anthropometry’, ‘Biochemical’, ‘Physiological’, ‘Psychological’, ‘Skills’, ‘Injury’, and ‘Genetic’. Publications cited within this review (1) contained relevant data concerning the testing of football players, (2) included football players, and (3) were written in English. Studies were excluded on the basis that they (1) did not contain relevant information on testing protocols and (2) were not peer-reviewed articles. Studies were not included/excluded based on the competitive standard of football players, the age category of players investigated/discussed, or the geographic location of study origin.

## 3. Anthropometric Testing

### 3.1. Anthropometry

The physiological and tactical demands of football dictate that elite players must possess athleticism and the ability to develop and maintain high fitness levels [2]. There is notable heterogeneity in body size among elite players [3], which can relate to positional allocation. For example, being taller can be advantageous to goalkeeping and to central defence and central attack where aerial duals are contested, though it is common to observe a range in stature within players who play the same position [4]. Thus, it is unlikely that an optimal anthropometric ‘profile’ for success in football exists based solely on stature and mass. Nevertheless, there is evidence that successful youth players’ are often taller and leaner than their less successful counterparts [5] and that average height, mass, and body mass index differ according to positional role among professional players [6]. Routinely measuring stature and mass in professional players is unlikely to have benefits but can be useful during adolescence when players mature at different ages and rates [7]. Specifically, players maturing earlier than their age-matched peers are typically taller and heavier [8]. Those players may be preferentially selected due to the advantages this confers to physiological attributes such as muscle strength due to a phenomenon known as the maturation-selection hypothesis [9]. Due to the grouping of youth players by chronological age, those born in the first quartile of the selection year also benefit from being physically advanced compared to players born later, which is termed the relative age effect [10]. To reduce the impact of these phenomena and improve selection opportunities, methods have been developed to estimate the chronological age at which young players undergo peak height velocity (PHV), referring to the period of fastest growth in stature during puberty [7], and to predict the final adult height of youth players. The maturity offset method uses a sex-specific regression equation to estimate years from PHV and requires measurement of player height, sitting height, and leg length [11], whilst the percentage of predicted adult height method considers the height and weight of the player in addition to their sex and the average of both parental heights [12]. These methods are popular and practical within football academies due to their non-invasive nature [13] but have limitations in their accuracy versus more advanced methods, discussed in detail elsewhere [14,15]. Nonetheless, regular collection of anthropometric measurements from youth players is commonplace in football academies for the purposes of monitoring player growth throughout adolescence, with particular interest among practitioners and researchers in whether regular monitoring of anthropometry can improve player development and reduce injury risk [16].

### 3.2. Body Composition

Whilst anthropometric measures in isolation may have limited utility, assessing body composition is common in professional football [17], where mean body fat levels of ~10% are typical in elite male players [18]. Excess body fat can negatively impact player mobility, power-to-weight ratio, total energy expenditure, and acceleration capacity [19,20]; thus, it is important for players to maintain appropriate fat mass. Where a player is injured and has lower energy expenditure due to reduced training load, it also is important to monitor body composition to minimise detrimental change (i.e., increased fat relative to lean mass) [21]. Detecting meaningful change in body composition requires valid and reliable methods, with four or five compartment methods considered the gold standard [22]. Laboratory methods such as underwater weighing, isotope dilution methods, magnetic resonance imaging, and dual X-ray absorptiometry (DXA) offer high precision but are expensive, difficult to access, and typically require substantial expertise to operate and interpret [22]. Whilst DXA scanning has become cheaper and offers greater accuracy than bioelectrical impedance or skinfold-derived estimates of body fat [23], the use of anthropometric measures (i.e., skinfolds) is considered an acceptable alternative when conducted by staff with kinanthropometric training [22]. Such practical and cost-effective methods mean body composition can help to quantify the effectiveness of training and/or nutritional interventions and can also be tracked over the competitive season(s) as part of holistic approaches to player monitoring [24].

## 4. Physiological Capacity Testing

The intermittent nature of football dictates that players must possess considerable athleticism, specifically aerobic capacity and the ability to produce the rapid, forceful contractions required to execute explosive movements [2]. Attributes relating to players’ fitness can differentiate between those at different competitive levels [25] and starting players from substitutes [26], highlighting the utility of fitness testing. Recently, elite football practitioners across 24 countries reported strength (85% of respondents), aerobic capacity (82%), and power/reactive strength (78%) as the most assessed physical capacities [27], with similar responses reported elsewhere [17]. The onset of pre-season was cited as the most common time to conduct fitness testing, and the primary approach for interpretation was to compare an individual player’s test results against their previous scores. Indeed, determining baseline fitness during pre-season helps monitor changes throughout the season, provides benchmarks to evaluate the effectiveness of training interventions, and establishes targets for injured players undergoing rehabilitation [17,28]. As with most football-specific characteristics, various testing protocols are available for the assessment of players’ physiological capacity.

### 4.1. Aerobic Capacity

Football requires excellent aerobic capacity due to the fact that energy provision during match play relies on aerobic metabolism [4]. Moreover, elite players routinely cover in excess of 10 km per match and must actively recover from intermittent explosive actions [2]. Maximum oxygen uptake (VO_2max_) is the most common marker of aerobic capacity, and it is long established that VO_2max_ in football players can vary according to playing standard and positional roles [29], with improvements in VO_2max_ corresponding to greater distances covered during match play [30]. Assessment of VO_2max_ can be conducted in laboratory settings or indirectly using field-based protocols. Laboratory-based VO_2max_ testing in footballers can be conducted via treadmill running to replicate sport-specific motion [31] and can involve collecting supplementary markers of exercise tolerance such as blood lactate, which can be used to determine lactate threshold and inform training prescription [31]. However, field-based assessments of aerobic capacity appear most favoured [27]. This likely reflects most field-based assessments of aerobic capacity being simple to conduct on the training pitch using minimal equipment, and the capacity to assess multiple players simultaneously. Indeed, the most cited aerobic fitness tests in a recent survey of practitioners were the 30-15 Intermittent Fitness Test (30-15 IFT) and the Yo-Yo Intermittent Recovery tests (YYIR-1 and YYIR-2) [27], which are pitch-based running tests allowing multiple players to be tested at a time. The 30-15 IFT involves 30 s of running interspersed by 15 s passive rest, with running velocity increased after every effort [32]. Similarly, the highly popular YYIR-1 and YYIR-2 tests involve shuttle running (2 m × 20 m) interspersed with active rest, with incrementally increasing running velocities directed via audible signal. Maximal aerobic capacity is then estimated based on maximal running speed or highest level completed for the 30-15 IFT and YYIR-1/2, respectively. Other methods used to assess aerobic capacity in football may include the Bosco Test, Multi-Stage Fitness Test, modified Probst Test, and running a specified distance within a set time or vice versa [27,33]. It is possible to collect blood lactate measures in field settings, but sampling from multiple players may require several support staff and has additional considerations for sample handling and storage. In the same manner that field-based sprint and jump tests are replicative of match actions, field-based tests for aerobic capacity lend greater specificity and, consequently, increased validity versus laboratory testing [34], potentially offering better insight into players’ ability to perform during competitive match play [31]. It is important to consider that field-based tests usually provide estimates of maximal aerobic capacity compared to the accuracy achieved by laboratory tests. However, field-based tests are generally capable of detecting changes in football-specific fitness, which is often of greater interest to coaches and players than specific data points.

### 4.2. Linear Speed

Key match outcomes are often dictated by acceleration and the ability of a player to cover distances faster than opponents. Testing linear speed is achieved by players sprinting maximally across a set distance, with the use of timing gates recommended to reduce measurement error. Testing of sprint ability typically involves 10-, 20-, and 30-metre distances [27], which is perhaps unsurprising considering match activity profiles report most sprints to last between 2 and 4 s over distances shorter than 20 m [2]. It is equally important to consider specificity for sprint tests, as players begin most in-game sprint actions whilst walking or jogging rather than from a standing start. However, sprint tests from a “flying” start appear to be conducted less than sprints from stationary positions [27], which may limit ecological validity [31]. Nevertheless, sprint testing represents a key component of any football testing battery and can inform the design of training interventions. For example, the relationship between maximal lower limb strength and sprint performance in elite footballers [35] suggests that combined strength and sprint training can enhance acceleration capacity, with repetition of the original sprint test determining the effectiveness of the intervention.

### 4.3. Muscle Strength

Muscle strength is critical to explosive actions involving muscular power, and high levels of strength can be associated with reduced injury risk [36]. Imbalances in muscle strength, on the other hand, have been recognised as an injury risk factor; thus, strength testing is routine in both youth and professional football. When assessing maximal strength, efficient tests that provide meaningful data without inducing undue fatigue or muscle damage upon players are desirable. Accordingly, simple isometric strength assessments, such as the isometric mid-thigh pull and adductor squeeze, have gained popularity within professional football and demonstrate high between-day reliability [27]. Both tests are time-efficient, easy to perform, and less fatiguing than traditional one- or three-repetition maximum tests using dynamic movements such as the back squat or bench press [37]. Another relatively new test is the Nordic hamstring exercise, which can be used as an assessment of eccentric hamstring strength or a screening tool for hamstring injury risk [38]. It remains common, where accessible, to establish player strength profiles such as hamstring-to-quadriceps (H:Q) ratios by measuring concentric and eccentric peak torque of the quadriceps and hamstrings via isokinetic dynamometry, typically during the pre-season period to establish player baselines and identify discrepancies between both muscle groups [39]. However, poor correlation of strength measured by Nordic hamstring testing with isokinetic testing suggests that different characteristics of muscle strength are captured by each test [39], and the value of isokinetic strength measures for predicting injury risk is debated [40,41]. A notable advantage of contemporary tests such as the isometric mid-thigh pull, adductor squeeze, and Nordic hamstring exercise is the development of specialist devices that are easily operated and often portable, meaning testing can be conducted in field- or gym-based environments within a short timeframe, as opposed to the time-consuming nature of isokinetic and/or repetition maximum testing across whole squads. Generally, it appears that laboratory-based assessments such as isokinetic dynamometry are being supplemented and/or superseded by gym- or field-based tests of isometric muscle strength, which also appear preferable to more taxing repetition maximum tests. Regardless of measurement protocol, the association of muscle strength with acceleration, movement velocity, jump ability, and sprint speed [35,42] justifies muscle strength as the most measured physiological characteristic in elite footballers [27].

### 4.4. Muscle Power

Whilst strength is a key component of power development [2], contraction velocity is required for the translation of maximal strength to power [43], meaning players’ muscular power cannot be assumed from strength testing alone. Dynamic movements involving rapid contractile activity (i.e., sprinting and/or jumping) also offer greater ecological validity than isometric or isokinetic strength tests by replicating force production in a manner specific to match play. This means such tests require little familiarisation for players [27], making it easier to sanction and implement frequent tests of power versus testing maximal strength or aerobic capacity [44] and contributing to favourable reliability [45,46]. Indeed, the most common tests of power in football settings are jump variations, including the Countermovement Jump and Squat Jump [27,44], with other tests of power and/or reactive strength reported by practitioners, including the single-leg countermovement jump, the drop jump, repeated jump tests, and hop variations [27]. The requirement to execute decisive and explosive actions during matches perhaps explains why more practitioners test for power and reactive strength than speed, agility, or repeated sprint ability (RSA) [27], though each of these attributes is influenced by the capacity to produce muscular power.

### 4.5. Repeated Sprint Ability

Single sprints are involved in key match actions; however, the activity profile of football intermittently requires the execution of repeated sprints within short timeframes [2]. Despite aerobic capacity being an important determinant of recovery between sprints and correlating with RSA [47], specific RSA testing can provide additional information to coaches on the intersection of aerobic capacity with muscular power. Protocols to assess RSA reported by practitioners typically involve 6 to 8 repetitions of distances ranging from 25 to 40 m per sprint, interspersed by rest periods lasting between 10 and 25 s [27]. This is not dissimilar to a validated protocol of Bangsbo [4] involving 7 m × 35 m sprints with 25 s recovery, which others demonstrated could discriminate elite from sub-elite youth and senior players [48,49]. Accordingly, testing players’ RSA can be included within a field-based testing battery as a time-efficient and practical method to aid selection decisions and monitor fitness. Nonetheless, it is curious to note that RSA was the least common (32% of respondents) of all physical capabilities assessed by practitioners in a recent study [27]. It is possible those practitioners prioritise strength, power, and aerobic capacity tests, perhaps due to time constraints with RSA tests requiring longer to complete than single-effort sprint tests or due to their awareness of the documented link between aerobic capacity and RSA, meaning they might infer RSA ability based on players’ performance in other tests.

### 4.6. Agility

The dynamic and unpredictable nature of match play requires players to have good agility for rapidly changing directions [50]. Agility combines strength and speed with balance and coordination, and valid tests of agility should incorporate fast and frequent directional change in a manner replicative of football [31]. Some consider agility tests the most appropriate field-based test for soccer performance because they discriminate elite footballers from non-athletes better than strength and power tests [48]. However, only ~50% of elite football practitioners report using tests of agility [27]. The 505 agility test developed in the 1980s remains the most popular method to assess agility in elite football [27], incorporating sprinting for 15 m, turning 180 degrees, and sprinting back for 10 m. A player’s time is recorded for 5 m before and after the turning point and can be performed twice to allow one change per leg [51]. A range of alternative agility tests is available, including the Illinois Agility Test, *t*-test, and Arrowhead Agility Test [27], with each test also requiring athletes to change direction rapidly through a standardised circuit. New football-specific agility protocols have been recently designed that incorporate both pre-planned and responsive directional change based on the addition of variable external stimuli [52,53], which may improve ecological validity.

In summary, practitioners testing football players’ physiological capacity appear to prioritise strength, aerobic capacity, and power. There are multiple testing protocols available for testing the various components of physiological capacity, many of which are shown to be valid with good inter-day reliability, allowing the detection of change across time. Where possible, time-efficient yet informative assessments that can test large numbers of players within field/gym-based environments can help practitioners limit player load and, if required, facilitate regular testing to identify trends in performance.

## 5. Biochemical Testing

The demands of modern football at the professional and semi-professional levels often involve congested fixture schedules and training cycles to prepare for competition [54]. In some instances, the time between matches is insufficient to achieve complete recovery [55], despite considerable efforts to manage player workloads [56]. During such periods, assessing recovery status is of great importance [57], principally because fatigue impairs physical performance and increases injury susceptibility [58]. Recovery following competitive football involves overcoming multiple internal perturbations, including exercise-induced muscle damage (EIMD), glycogen depletion, oxidative stress, altered immunity, and proinflammatory status [59,60]. Accordingly, it may be useful to supplement field-based physical capacity tests that screen for decrements in muscle strength and power with the assessment of biochemical parameters [57]. The assessment of biochemical markers typically involves collecting blood, urine, and/or saliva, with the aim of providing an objective measure of players’ internal load [59]. It is possible to separate prospective biochemical markers based on their physiological function, such as those relating to EIMD, oxidative stress, immune state, inflammatory response, and endocrine response [60].

### 5.1. Muscle Damage Markers

Muscle damage occurs during football through intense eccentric actions [61] and negatively affects muscular power, as evidenced by impaired jump and sprint performance [62,63]. Consequently, intracellular proteins are released by skeletal muscle into the bloodstream, highlighting the potential of EIMD screening via blood sampling. An analyte often measured for assessing EIMD is serum creatine kinase (CK), an intracellular enzyme that increases substantially in serum following eccentric contractions [64] and is elevated post-match in football players [59]. Performing unaccustomed eccentric exercise is associated with a higher EIMD response, meaning it may be particularly useful to test CK levels when players return to intense training or competition following periods of rest or reduced intensity, such as the off-season or a period of injury-induced inactivity [57,65]. However, serum CK demonstrates high inter-individual variability [66], and several potentially confounding variables, including ethnicity, genetic factors, sex, and training status, mean that adopting individualised reference values is critical for reliable CK player monitoring [67]. This approach would also account for the fact that football players typically have higher CK values than non-athletes, which is likely due to the volume of contractile activity from football activity [68]. Serum lactate dehydrogenase (LDH) can also indicate muscle damage, with increases following eccentric exercise in a manner similar to CK [69]. The LDH enzyme functions to catalyse the conversion of pyruvate to lactate and is present in most tissues. However, unlike CK, this means that LDH can enter the circulation from multiple origins, making measurement of muscle-specific LDH from serum challenging [59]. Myoglobin represents another muscle-specific protein known to leak into the circulation due to EIMD in football players but may be of limited value because levels return to baseline after 24 h [70], falling short of the typical time course for recovery from EIMD. It has also been suggested that assessment of the enzymes alanine transaminase (ALT) and aspartate transaminase (AST) might be used to assess EIMD in footballers because both are released from skeletal muscle after intense exertion [71]. However, a limited number of studies are available, and those studies present inconsistent results, meaning definitive evidence on the time course of ALT and AST following football activity is lacking. Consequently, CK is currently the most evidence-based EIMD biomarker that may be used for player monitoring.

### 5.2. Inflammatory Markers

The onset of EIMD and subsequent release of intracellular proteins, including CK, triggers a substantial inflammatory response and release of inflammatory markers [38]. This inflammatory response initiates the natural repair and remodelling of skeletal muscle and can vary in duration depending on the volume and intensity of the preceding exertion [72]. Initially, the cytokines interleukin-6 (IL-6) and tumour necrosis factor alpha (TNF-α) and the acute-phase protein C-reactive protein (CRP) trigger an inflammatory state [73]. Serum IL-6 appears to be increased immediately post-match but returns to baseline after 24 h [74,75], suggesting IL-6 measurement may only reflect the initial inflammatory response as opposed to being a reliable marker of overall recovery. The release of IL-6 contributes to the secretion of additional proinflammatory cytokines, such as TNF-α and interleukin-1-beta, which have each been shown to increase acutely following a football match [74,76]. The acute-phase inflammatory response following acute and strenuous exertion transiently increases CRP [77], a hepatically synthesised protein shown to peak in the circulation around 24 h post-match and return to baseline at 72 h [75,76]. Consequently, some suggest CRP as a prospective marker for monitoring players’ inflammatory status in the days following a match [59], perhaps because the time course of CRP better reflects the typical pattern of EIMD than that of IL-6 and TNF-α.

### 5.3. Immune Markers

The initial release of cytokines following exertion is known to contribute to an immune response [78], where a number of analytes can provide an indication of an athlete’s immune status. Like inflammation, it should be noted that the immune response represents part of normal physiological repair and regeneration following EIMD [79], but also that it can be challenging to differentiate increased immunosurveillance from suppressed immune function based on biomarker sampling [80]. Nevertheless, some suggest immune function may be impaired following strenuous exertion, prompting interest in the monitoring of football players’ immune status. Temporary changes in white blood cell (WBC) counts have been observed for up to 48 h post-match and may be especially pronounced during congested fixture periods [65,76,81]. However, multiple investigations of WBC immediately after and in the days following competitive football have investigated a range of subtypes, including lymphocytes, monocytes, and neutrophils, which exhibit variable patterns (increase or decrease) in the days following competition (see review by Pèrez-Castillo et al. [59]).

Immunoglobulins represent an alternative marker of immune status and may also be altered by congested football schedules [82]. Immunoglobulin A is the most abundant, and salivary immunoglobulin A is commonly measured in studies of athletes, likely due to ease of sampling. However, the available evidence concerning immunoglobulin activity in response to intense exercise is inconclusive [83], with studies reporting inconsistent findings based on different methodological approaches. Accordingly, we propose more evidence is warranted before immune markers such as WBC count and immunoglobulin levels are used to monitor player immune status and recovery.

Recently, circulating cell-free DNA (cfDNA) has been highlighted as a damage-associated molecular pattern that is increased during competition compared to pre-competition in rowers [84]. One study of professional footballers documented significantly elevated cfDNA the day after competitive matches and the correlation of cfDNA levels with training data [85], suggesting potential utility as a biomarker for load monitoring. However, more studies are required before the value of cfDNA monitoring can be judged appropriately.

### 5.4. Endocrine Markers

Exercise represents an external stressor capable of upsetting internal homeostasis, whereby endocrine hormone release follows as a means of adjustment to cope with the stimulus [86]. Following football matches, endocrine markers may be affected for up to 72 h [60]. Cortisol is the primary steroid hormone linked to the stress response and is involved in mediating energy provision [87], with the rationale for monitoring player cortisol levels arising from suggestions that cortisol contributes to impaired sporting performance as a consequence of increased catabolism [88]. Indeed, increased cortisol is observed immediately and up to 48 h post-match [76,81,89] but not in all studies [75], and evidence concerning cortisol levels during congested periods remains conflicting [76,90]. Testosterone is another steroid hormone with potential for testing in football players. In contrast to cortisol, testosterone is an anabolic hormone implicated in protein synthesis and can negate the catabolic action of cortisol [91]. Elevated free and total testosterone levels are reported immediately post-match by some [75,81] but not others [76]. Some suggest the testosterone/cortisol (T/C) ratio may be useful to measure the balance between anabolism and catabolism and to indicate the level of wellness/fatigue as a means of avoiding overtraining [60,66]. Indeed, a decrease in the T/C ratio has been associated with reduced fitness test performance [92], fatigue [93], and congested fixture schedules [94]. Despite these observations, the T/C ratio has been deemed unreliable for indicating the balance between anabolism and catabolism in football players recovering from match play [59,75]. It is important to note that a number of factors may also influence changes in cortisol and/or testosterone levels pre- to post-match, including circadian rhythm, hydration status, sex, match context, and match result, and that strict control surrounding sampling procedures also presents a notable challenge to reliable hormone monitoring in footballers [59].

### 5.5. Oxidative Damage Markers

Oxygen metabolism during muscle contraction generates free radicals because of enzymatic reactions [95], and perturbations in the balance between oxidative and antioxidative systems can lead to a pro-oxidative state characterised by the oxidation of cellular components, promoting inflammatory mechanisms and cell death [96]. These perturbations may intensify the impact of EIMD and may also be aggravated by the onset of EIMD [97,98], with oxidative stress contributing to reductions in muscle force [99]. For this reason, and because of the link between oxidative stress and inflammation, circulatory oxidative molecules have emerged as candidates for monitoring oxidative stress in athletes [95]. Indeed, football match play promotes oxidative damage, and changes in oxidant and antioxidant markers are observed immediately post-match for up to 72 h [60]. Oxidant markers include lipid peroxidation by-products such as malondialdehyde (MDA), 8-hydroxy-2-deoxyguanosine (8-OH-Dg-8), whilst antioxidant markers include catalase (CAT), superoxide dismutase (SOD), glutathione peroxidase (GPX), glutathione (GSH), glutathione disulfide (GSSG), and uric acid (UA) [59,60]. Lipid peroxidation can also cause significant cell damage and can be quantified using the formation of plasma thiobarbituric acid reactive substances (TBARS) [59]. The potential appeal of testing oxidative stress markers in footballers may be influenced by observations in multiple studies that both MDA and TBARS increase immediately post-match for up to 72 h [76,100,101,102], yet the methods for assessing these markers have been criticised [59]. Regarding markers of antioxidant activity, plasma UA levels are reported in some studies to increase substantially following a match and to reflect total antioxidant capacity [100], whilst others found no change [88]. In contrast, the level of GSH and the GSH/GSSG ratio each decrease post-match and might indicate oxidative stress related to the inflammatory response [76,100], though the duration of this alteration is yet to be determined. The GPX enzyme catalyses the oxidation of GSH to GSSG when free radicals are present, with other antioxidant enzymes such as CAT also investigated following match play. Increased CAT activity is typically observed for a short period before returning to baseline in less than 24 h [76]. Evidence surrounding GPX activity is less clear, with increases [76,101] and decreases [102] reported in the 24 to 72 h post-match. Taken together, it is unclear how long perturbations in oxidative markers persist following match play and whether congested periods exacerbate this response despite consistent evidence of alterations for up to 72 h. This suggests further evidence is required before routine assessment of oxidative status in footballers is undertaken.

Together, a range of biochemical analytes has been proposed as candidates for monitoring player health and recovery status in football. However, it is not always possible to collect biological samples non-invasively, and many challenges exist regarding standardisation or protocols for collecting, storing, and analysing samples. Furthermore, there is very limited evidence demonstrating the efficacy of biochemical screening in identifying the onset of pathology, which questions the necessity of biochemical testing when other methods for assessing player wellness are available. Clubs and practitioners may opt to periodically perform biochemical screening of players, but less invasive assessments of physiological capacity and perceived recovery may offer more practical choices for regularly monitoring recovery status within large playing squads and may also provide better indicators of fatigue and/or impaired recovery.

## 6. Psychological Testing

Assessing the psychological characteristics of football players can be undertaken at various age levels to address different objectives. In talent identification situations where the aim is to recognise those with the potential to develop into elite players, understanding factors such as motivation, coping with stress, resilience, commitment, discipline, and concentration may be advantageous [103]. Indeed, problem-focussed coping behaviours, social support seeking, goal commitment, discipline, and motivation are documented as predictors of career success in footballers [104,105]. Nevertheless, extensive research on the utility of psychological factors during talent identification is lacking [103], making it difficult for coaches to employ standardised or evidence-based procedures. Another use of psychology-based measures in the football environment is the use of psychometric tools to assess player wellbeing, primarily in the contexts of stress, fatigue, and recovery [106]. Specifically, the early detection of subjective fatigue and suboptimal recovery can be an important preventative step in the avoidance of overtraining. This reflects evidence that increased training load, fatigue, and/or an imbalance between training and recovery can impact the psychological response of football players to training [107,108,109], including poor concentration, negative feelings, and mental fatigue [110]. Consequently, many suggest that psychometric monitoring be used to maintain optimal performance in footballers, with several methods available.

### 6.1. Mood

The profile of mood states (POMS) is a validated questionnaire used to assess emotional states characterised by positive or negative feelings [106]. The POMS questionnaire has demonstrated the relationship between disturbed mood state (i.e., increased feelings of anger and depression) and reduced performance in young elite players [111], with similar evidence of heightened negative mood states in professional players undertaking intense training schedules [112]. The questionnaire is simple to administer and can be repeated to detect changes in mood [106], meaning it can be incorporated within a holistic approach to player monitoring. The Hooper Index (HI) [113] incorporates mood with psychophysiological indices by asking athletes to rate their previous night’s sleep, stress, fatigue, and delayed-onset muscle soreness, and may be particularly useful for assessing subjective recovery [107]. Indeed, these factors can negatively affect athletic performance, either in isolation or in combination [108,113]; thus, the validity of the HI is reflected by the observation that scoring is associated with both performance measures and training load intensity [114].

### 6.2. Perceived Recovery

A similar psychometric tool to the HI is the Total Quality of Recovery (TQR) scale, which is also validated to quantify recovery status and is deliberately formatted in a similar manner to the perceived exertion scale to reflect the relationship between training and recovery [115]. The TQR assesses athletes’ perception of recovery by grading activities undertaken in the preceding 24 h that contribute to optimal and accelerated recovery, including nutrition/hydration, sleep/rest, relaxation/emotional support, and stretching/active rest. Like HI, TQR scores are predictive of football performance and are sensitive to increases in training load [116], making TQR a suitable candidate for daily player monitoring.

A further assessment tool designed to prevent overtraining in sportspeople is the Recovery-Stress Questionnaire for Athletes (RESTQ-Sport) [117], which is capable of detecting changes in stress and recovery over short timescales [118,119]. In football, the RESTQ-Sport shows utility in identifying elite youth players at risk of overtraining [120] and has also demonstrated that both general and emotional stress are higher in youth players suffering minor illnesses, leading to temporary absence from training and/or match play [121].

Overall, non-invasive and non-fatiguing methods such as the HI, TQR, or RESTQ-Sport appear favourable for coaches to monitor players by complementing other tests, such as those of physical capacity, to determine player readiness. The incorporation of psychophysiological indices within these tools is likely to reflect holistic recovery better than tools assessing psychological state only, such as the POMS questionnaire, though the latter may have utility in some situations.

### 6.3. Perceived Return-to-Play Readiness

Beyond acute recovery and readiness to perform, psychological factors also make an important contribution to a player’s recovery from injury [122]. This can be particularly relevant as a player nears return-to-play [123], with suggestions that regular screening throughout rehabilitation may help identify adverse psychological responses to injury [124]. Several psychological screening scales related to injury are available, including the Re-Injury Anxiety Inventory (RIAI) [125], the Injury Psychological Readiness to Return to Sport (I-PRRS) scale [126], the Anterior Cruciate Ligament-Return to Sport after Injury (ACL-RSI) [127], and the Psychological Readiness of Injured Athlete to Return to Sport (PRIA-RS) scale [128]. However, some suggest that many of these scales have limited utility in footballers. For example, the I-PRRS only considers the athlete’s level of confidence rather than probing other emotions; the RIAI is only concerned with feelings of anxiety concerning re-injury risk, and the ACL-RSI relates to ACL injuries only [129]. Consequently, the PRIA-RS was designed to detect increased re-injury risk as an athlete approaches return-to-play by assessing multiple factors, including perceived rehabilitation success, mood, confidence in physical capacity, re-injury anxiety, motivation to return to competition, and emotional readiness [130]. The PRIA-RS has been validated in a cohort of professional footballers [129], demonstrating the capacity to determine players’ psychological readiness to return to play and the potential utility of the PRIA-RS to reduce injury recurrence.

Taken together, it may be advantageous to use validated psychometric screening tools to supplement physiological data when assessing both player recovery and readiness for return-to-play from injury. Evidence in football players suggests the Hooper Index, Total Quality of Recovery scale, or RESTQ-Sport may perform best when monitoring recovery during congested periods, whilst the PRIA-RS scale appears suitable for determining individual readiness for return-to-play.

## 7. Injury Risk Testing

Injury is the primary factor contributing to player availability in professional football [131] and is consequently linked to team success [36]. For youth players, injury negatively impacts the likelihood of reaching the first team [132], and there is a general consensus that previous injury is a risk factor for subsequent injury [133]. Therefore, considerable attention is afforded to injury prevention and load management, with a significant body of research investigating injury in football. Lower limb injuries to the thigh, knee, and ankle are most common in professional and academy players [134,135], which has directed the design of many prospective screening tests for identifying players’ injury risk. Similar to assessing other attributes, the development of simple, low-cost tests that can be applied to squads of players in field or gym environments is desirable [136].

### 7.1. Movement Competency

The Functional Movement Screen (FMS) is reported as the most common injury risk screening test among elite professional teams [137] and the third most common in English academies [138]. However, three systematic reviews have scrutinised the use of FMS to predict injury, with one supporting its’ use [139] and two concluding poor concordance between FMS scoring and injury [140,141]. The FMS assesses multiple movement tasks by visual observation based on standardised criteria [142], and some suggest a lower composite score as indicative of greater injury risk. In youth football players, FMS did not predict subsequent injury [143], with a football-specific systematic review recommending further investigation of the validity and reliability of the FMS to identify players at risk of injury [133].

### 7.2. Strength, Flexibility, Range of Motion

Another popular screening tool amongst professional clubs is isokinetic dynamometry to calculate the H:Q ratio [137,144], primarily for the purposes of hamstring injury prevention. Whilst commonly used, there is inconclusive evidence to support a link between isokinetic testing and hamstring injury in football [133,145,146], despite demonstrated associations in other sports [136].

Joint range of motion (ROM) assessment, most often performed using goniometry, was the most commonly used (76% of respondents) injury risk screening tool in a survey of English football academies [138] and has been investigated in relation to various lower limb injuries. Decreased ROM in hip abduction has shown reliability in screening for groin strain injury risk in footballers [147] and may be predictive for lower limb injury in general [136]. Similarly, decreased ROM during hip flexion was shown to predict hamstring injury risk in elite players [148], yet ROM during pronation, supination, and dorsiflexion was not predictive of ankle injury [149]. In elite football, flexibility is cited among the most popular tests to screen for injury risk [137] and forms a key component of youth coaches’ injury prevention efforts [138]. Flexibility of the hamstrings is commonly assessed and can be conducted using tools such as the Toe-Touch Test, Sit-and-Reach Test, and Thomas Test. In footballers, hamstring flexibility was not associated with ankle injury risk in footballers [149], with conflicting results concerning a link to hamstring injury risk in Australian rules footballers [150,151]. Others suggest deficits in passive and active flexibility should be considered a hamstring strain risk factor in football [152] based on assessments that included elements of the FMS.

Like the FMS, the Soccer Injury Movement Screen (SIMS) assesses the quality of multiple movements selected specifically to reflect the most common sites of football injuries, including the knee, ankle, and hip [153]. However, in non-elite players, the SIMS was not associated with injury either as a composite score or when considering individual components, demonstrating an inability to categorise players by their projected injury risk [154].

### 7.3. Balance

The Star Excursion Balance Test (SEBT) is another example of a low-cost and time-efficient tool that assesses dynamic postural control during single-leg balance [155]. The SEBT offered predictive ability for lower extremity injury in basketball [156] and recently demonstrated association with lower limb injury and lower back pain in amateur footballers [157]. However, others suggest that a history of ankle injury may affect performance on the SEBT in footballers [158], and there is limited evidence to confirm or dispute the efficacy of the SEBT as an injury screening tool in professional football. The Y-Balance Test (YBT) is a derivative of the SEBT incorporating only the anterior, posteromedial, and posterolateral components [159]. One study in a mixed cohort of professional and non-professional players found that the YBT could identify players susceptible to injury [160]. In contrast, another study of elite youth players investigating only the anterior YBT component urged caution due to weak associations with injury occurrence [161]. Indeed, the YBT was not among the most commonly used injury screening tools in a survey of English professional academies [138], which may reflect a lack of supporting evidence.

### 7.4. Landing Mechanics

The Landing Error Scoring System (LESS) is a field test designed to screen for risk of injury to the anterior cruciate ligament (ACL), one of the most severe football injuries for time loss, chance of re-injury, and risk of degenerative knee conditions in later life [162,163]. The test aims to identify errors during landing, with higher scores indicative of greater injury risk. Scoring on the LESS was not associated with ACL injury risk in a cohort of collegiate athletes from multiple sports, including football [164]. However, LESS scores were higher for injured players than non-injured players in a cohort of male and female youth footballers [165], suggesting the potential utility of the LESS to predict ACL injury risk in football. As is the case for many injury screening tests, further evidence is required to confirm the validity and reliability of the LESS, particularly in professional players.

### 7.5. Biomechanics

The LESS represents one of several approaches based on the concept of biomechanical assessment. Given the prevalence of hamstring strains and injuries to the ACL in football [134,135], efforts have been made to develop valid and reliable screening tools aimed at preventing these injuries using biomechanical analysis. Hamstring strain injuries typically occur during sprinting [166] and may be related to sprint mechanics [167]. It is postulated that hamstring injury risk may be heightened by fatigue, leading to a decrease in hamstring contractile strength and altered sprint kinematics [168]. Sprint mechanics have traditionally been assessed using three-dimensional motion capture, which is often time-consuming, expensive, and limited to laboratory environments where assessing teams of players is challenging. For this reason, qualitative movement screening tools such as the Sprint Mechanics Assessment Score (SMAS) [169] have been developed. The SMAS uses slow-motion video capture to rate 12 items related to movement quality during sprinting using dichotomous scoring, where a higher score indicates poor technique [169]. The tool demonstrated good reliability in male and female players assessed during 35-metre sprints and may be useful within a battery of injury screening tests. Similarly, the LESS and alternatives such as the Cutting Movement Assessment Score (CMAS) [170] screen movement mechanics related to ACL injury.

A prominent risk factor for ACL injury is knee valgus during landing and cutting [171]; thus, a number of protocols are available to assess the lower limb mechanics of players during these situations, with many investigated in youth players. This is partly because periods of rapid growth, such as surrounding PHV, are associated with alterations in landing mechanics that might increase injury risk [172]. Similarly, asymmetry between limbs is also recognised as a risk factor for ACL injury in youth players, prompting the development and use of tests to assess lower limb mechanics during landing. The tuck jump assessment involves repeated tuck jumps being recorded and visually assessed against set criteria, with knee valgus estimated by measuring frontal plane projection angles (FPPA) between the centre of the hip, knee, and ankle joints [173]. In male youth players, the tuck jump is a reliable test for assessing the presence of knee valgus [174] and, thus, may be particularly useful for screening landing mechanics in young players. Additional movements studied in the literature single-leg jumps and hops in different directions, whereby the focus is placed on the ability to stabilise the lower limb upon landing [174], and may be used to regularly screen young players in field-based settings to monitor changes in landing mechanics across time.

Despite a range of tests purported to identify athletes at risk of sporting injury, some still require validation in cohorts of football players. Several screening tools have demonstrated an association with either injury incidence or injury mechanisms in football cohorts, yet experts indicate that the ability to predict future injury using screening tests is an unrealistic expectation that is unsupported by current evidence [175,176]. Importantly, it has been proposed that screening should primarily be used to benchmark players’ musculoskeletal function rather than to test or predict their injury risk [176]. Nevertheless, many tests often used as injury screening tools demonstrate validity for their original purposes, such as assessing movement competency, meaning they may still offer benefits within a holistic approach to injury prevention, particularly where movement mechanics are recognised risk factors. Despite the inability to confidently predict injury occurrence in football, the prevention of non-contact injuries may be aided by developing sufficient physical qualities in football players from a young age, including muscle strength, flexibility, and range of motion, and by managing player loads to allow sufficient recovery during congested periods. Injury prevention strategies should involve screening and monitoring undertaken by a multidisciplinary team of practitioners, including (but not limited to) medical staff, coaches, and sports scientists, who can each provide complementary insight and information.

## 8. Football-Specific Skills Testing

Beyond the importance of athleticism and physiological capacity, the ability to successfully execute skill is the principal aspect of football match play [103,177]. Indeed, a survey of staff from 29 professional academies worldwide documented technical skills as the most important attribute in youth players [178]. Furthermore, those tasked with player recruitment describe first touch and the ability to strike the ball amongst the most desirable player attributes [179]. This has been appreciated for many years, as exemplified in 2000 by the development of the F-MARC test battery designed to replicate and test a range of football-specific skills and fitness indicators [180]. More recently, several technical skills from the F-MARC battery were described as reliable for assessing youth players, including dribbling, passing, shooting, and heading [181]. This is in agreement with a study of German youth players demonstrating kicking skills and dribbling as the most important technical skills for predicting whether under-12 players remained within the same academy three years later [182] and prior evidence that the performance level of such skills discriminates elite from sub-elite youth players [183]. Accordingly, tests or testing batteries that assess technical skills appear favourable for talent identification and/or evaluating the development potential of existing players.

A recent scoping review of skill-based assessments employed in youth football found field-based assessments as the most frequently used, with the majority of protocols assessing dribbling speed and/or accuracy when passing or shooting [184]. It is noteworthy that those authors identified 226 different skill tests used to assess youth players, suggesting there is a lack of consensus regarding the optimal method of skill assessment. Nevertheless, there are some protocols for assessing specific skills that have received more attention.

### 8.1. Dribbling Performance

For dribbling, the UGent Dribbling Test [185] requires players to run as fast as possible through a circuit involving left and right turns, one attempt without the ball and a second with the ball. This test is shown to be reliable in adolescent players [185], and performance in the test correlates with how coaches rank players, particularly midfielders [186]. The Shuttle Sprint and Dribble Test and the Slalom Sprint and Dribble Test were designed for assessing hockey players [187] but have both been adapted for football, demonstrating good reliability [188]. The shuttle variation involves three 30-metre sprints without a ball and three with a ball, with three 180-degree turns during each sprint. In contrast, the slalom variation requires players to slalom laterally between cones for a 30-metre distance, once with a ball and once without. One study of Dutch youth players found that those performing well on these tests were more likely to be retained in talent development programmes [189], whilst another reported that an adapted version of the Shuttle Sprint and Dribble Test (three sprints with the ball) could discriminate youth players who became professionals from those who did not [190].

### 8.2. Passing and Shooting Accuracy

The popular Loughborough Soccer Passing Test (LSPT) is a valid and reliable assessment incorporating passing, dribbling, ball control, and decision-making [191,192]. Players are required to complete 16 passes as fast as possible based on instructions from testers, with penalties deducted for mistakes [191]. A higher score indicates superior performance, and the LSPT can discriminate players of different abilities, offering potential utility for talent identification [193]. The Loughborough Soccer Shooting Test (LSST) was developed at the same time and is also valid and reliable for assessing shooting performance, helping discriminate elite from non-elite players [191]. The LSST incorporates passing and ball control before players shoot toward targets on a regulation-size goal, aiming to score as many times as possible from a trial of 10 shots (five on each foot). The LSST also distinguishes between playing levels and detects changes in shooting performance [194], indicating potential use for both talent identification and assessing the efficacy of interventions to improve shooting ability. More recently, the 356 Soccer Shooting Test (356-SST) also demonstrated sufficient sensitivity to distinguish between playing levels in adult players [195]. The 356-SST assesses shooting accuracy, shooting quality, and ball velocity from shots taken from a two-step run up. Those authors suggest that the 356-SST focusses more on shooting proficiency by eliminating potential interference arising from the involvement of other skills, such as those included in the LSST [195]. Whilst further evidence is required to confirm the validity, this might make the 356-SST more ecologically valid for specific scenarios such as dead-ball situations (i.e., free-kicks or penalty kicks), and the LSST more ecologically valid for assessing shooting scenarios that arise from open play.

### 8.3. Match Play Simulation

It is important to consider that whilst specific tests of football skills may help predict future success and distinguish between playing levels, coaches perceive in-game scenarios with high importance [196]. Isolated skills tests, even when part of a testing battery, remove many of the dynamic and unpredictable elements of match play, meaning simulated methods of assessment, such as small-sided games, may offer additional insight [184]. Indeed, authors commenting on other team sports suggest match play is the most valid method of skill evaluation and laboratory testing the least valid, with field-based tests (simulated superior to isolated) offering an intermediate level of precision [197]. Where possible, testing batteries for skill execution that incorporate dribbling, shooting, and passing could be supplemented by analysis of small-sided game performance. Nevertheless, we propose that, in general, testing of specific football skills is most suitable in younger players for the purposes of talent identification and/or development due to the fact that professional players will have already demonstrated sufficient skill in match play situations to justify reaching the elite level.

## 9. Genetic Testing

The popularity of football has prompted interest in the genetic factors separating elite players from non-athletes, whether specific variants are associated with football-specific physiological capacity, and whether there may be a genetic susceptibility to injury [198]. Indeed, athletic performance represents the interaction between genetic and environmental factors, with athlete status being approximately 66% heritable [199]. By definition, athlete status incorporates the heritability of specific phenotypes paramount to football performance, including aerobic capacity, muscle fibre composition, and muscle strength [200,201,202]. Specifically, it is sequence variation, such as single-nucleotide polymorphisms (SNPs), within or close to genes that can affect the proteins produced by those genes and potentially impact tissue characteristics [203]. To date, most studies of genetics in football have studied a single or small number of variants in modest sample sizes of youth or elite players, with only a handful of genetic variants associated with football-related phenotypes in more than one study.

A recent review concluded that only six genetic variants have robust evidence of an association with becoming an elite footballer [198]. These variants were discovered by candidate gene studies, whereby genes with known function are hypothesised to impact physiological parameters. The variants associated with elite football status are linked to blood pressure regulation (*ACE*, *AGT*) [204,205], muscle fibre composition (*ACTN3*) [205,206], lactate transport (*MCT1*) [207], nitric oxide synthesis (*NOS3*) [208], and fatty acid oxidation (*PPARA*) [205,208]. Similarly, six SNPs have robust evidence of association with football-specific physical performance, discovered by conducting sport-specific tests in footballers and evaluating whether performance differed according to their genotype (genetic profile). Associations were reported with jump height (*ACTN3*, *COL5A1*) [208,209], aerobic capacity (*ACTN3*) [209], and sprinting/agility (*AMPD1*, *BDNF*, *COL2A1*, *COL5A1*, *NOS3*) [208], but require independent replication before being considered reliable predictors of performance. Some of those SNPs associated with football status and/or performance are among seven SNPs associated with football injuries. These include associations with the occurrence and/or severity (recovery time) of all injury types combined and with specific types of injury, including soft tissue, skeletal muscle, knee (ACL and MCL), ankle, and hamstring. The SNPs associated with injury are found within genes that encode for proteins affecting muscle fibre composition (*ACTN3*) [209,210,211,212,213], structure and function of collagenous soft-tissue (*COL1A1*, *COL5A1*, *EMILIN1*) [211,214,215,216,217,218,219], inflammation (*CCL2*, *IL6*) [211,218,219,220], and extracellular matrix degradation (*MMP3*) [211,220]. However, there is still inconsistent evidence regarding the association of these SNPs with injury in football, meaning these findings require independent replication in considerably larger samples. Furthermore, the hypothesis-driven approach used to date omits the ability to discover unknown genomic regions associated with football-related phenotypes, with hypothesis-free genome-wide association studies required to address this problem. The most significant genetic variants associated with football player status are summarised in Table 1. This table also includes the most robust genetic markers linked to other sport-related traits (such as endurance, power, strength, coordination, and spatial ability) [221,222], which should be tested for significance in football players in future studies.

In the future, the evidence base surrounding sports genomics is likely to improve and could facilitate the inclusion of genetic information to complement traditional approaches for talent identification, training prescription, and injury prevention [267]. However, the current scientific consensus is that there is insufficient evidence to underpin a rationale for genetic testing in sport [268,269,270], despite the perception of athletes and coaches that such information could be useful [271]. To date, it has proved challenging to recruit the sample sizes needed to robustly investigate football-specific traits in elite players, which will require large, concerted efforts involving several clubs and/or national teams [272,273,274,275]. Nevertheless, coaches, practitioners, and players should recognise genetics as a non-modifiable factor that contributes to footballing ability, but also that insufficient evidence is available for genetics to inform practice.

## 10. Practical Considerations

The choice of testing protocols in football is influenced by several factors, including cost, time constraints, staffing, and perhaps most importantly, the rationale testing in the first place (Table 2). Assessment protocols that can be incorporated regularly by football clubs with minimal disruption to players’ training and competitive schedules are likely to be favoured by coaches and practitioners, and the ability to test multiple players in on-field settings is highly desirable.

For talent identification and/or when working with youth players, assessing football-specific skills is of high importance, particularly dribbling, passing, and shooting. It may also be beneficial to assess players’ psychological traits due to the association of specific qualities with career success and to evaluate physical capabilities such as speed and agility. Anthropometric testing of youth players can provide an indication of physical development in order to limit the influence of the maturation-selection hypothesis and relative age effect and may have some utility for positional allocation. There is still debate surrounding the degree to which biological maturation, which can be estimated using anthropometry, impacts injury risk. Biochemical testing in youth players has limited merit, particularly because a key justification offered for such tests is the monitoring of fatigue and recovery. Youth players generally play fewer matches and compete at a lower intensity than their professional counterparts, which may limit the likelihood of cumulative fatigue, and ethical reasons may also preclude biological sampling in younger age categories. There is no evidence to support the use of genetic testing for talent identification at this time.

For established, professional teams where financial and staffing resources are often superior to academy environments, the main constraint surrounding testing is likely to be time. In professional and elite academy settings, communication and information sharing between medics, coaches, sports scientists, physical trainers, nutritionists, and other staff conducting individual testing are likely to enhance the management of player wellness and, ultimately, improve the effectiveness of testing. Where possible, additional assessment of factors, including dental health, nutritional status, and quantitative data on sleep, may further enhance the holistic understanding of player health. Considering the testing domains discussed within this review, skills testing may provide less insight for professional athletes than when assessing younger players, principally because those who have already achieved professional status are likely to have evidenced significant skill in match play situations to reach that level. Anthropometric testing can be used periodically to monitor body composition, particularly following periods of scheduled or enforced absence. Similarly, assessing players’ physical capacity after such periods can provide valuable information on individual and squad benchmarks for attributes such as muscle strength, aerobic capacity, power, and linear speed. Periodic repetition of these tests throughout the season can help identify training adaptations and the onset of fatigue. Regular psychometric screening can provide practitioners with additional insight into players’ subjective perceptions of fatigue and recovery to assess readiness and avoid overtraining, as well as supporting return-to-play after injury. Practitioners may wish to use injury screening tests but should be cognisant that most field-based protocols offer valid measures of movement competency rather than the ability to predict injury. The use of biochemical markers to complement tests of physical and psychological state demonstrate the potential for avoiding excessive fatigue, but test reliability is heavily influenced by the ability to standardise collection procedures. Finally, suggestions that genetic information be used to inform training prescription and/or determine injury risk in football are not supported by current evidence and are consequently not recommended.

## 11. Limitations

Whilst we have attempted to provide a comprehensive overview of the key testing domains in football, we recognise some limitations of our approach. Firstly, our narrative review is designed to provide a summary-level discussion of the various testing domains and most popular tests documented within the peer-reviewed literature. However, this may not capture additional tests that may be used in applied practice, either because these are used less frequently and/or may not be reported widely in the available peer-reviewed literature. Secondly, the narrative review approach allows a summary across a spectrum of testing domains but does not systematically review the evidence in support of the reliability and validity of specific testing protocols. However, a systematic review of all testing domains would be a considerably large body of work, and we highlight the existing availability of systematic reviews focussed on specific testing modalities. Finally, we acknowledge that most of the research described in our review provides data on male football players. Consequently, we highlight the urgent need for studies confirming that these testing domains and protocols have the same relevance, reliability, and validity in female players.

## 12. Conclusions

The present narrative review identifies several types of testing in football, including anthropometry, physiological capacity, biochemical parameters, psychology, skill execution, and injury risk. In isolation and/or combination, and dependent on player age or competitive status, protocols within these domains are often used for talent identification, assessing physical qualities, quantifying the efficacy of training interventions, and monitoring player fatigue and recovery status. Test selection and justification typically relate to cost, time constraints, and the ability to incur minimal disruption to players’ physical or psychological state. Indeed, short-duration, low-cost field-based assessments requiring minimal equipment that can be used to assess multiple players simultaneously appear favourable to football practitioners.

## Figures and Tables

**Table 1:** The most robust genetic markers for football player status and other sport-related phenotypes.

Gene	Full Name	Polymorphism	Favourable Allele	Phenotype	References
*ACE*	Angiotensin I converting enzyme	I/D	D	Football player status; sprint performance of football players	[204,205,223]
*ACTN3*	Actinin alpha 3	rs1815739 C/T	C	Football player status; CMJ performance of football players; power; testosterone levels	[205,206,209,224,225]
*ADRB2*	Adrenoceptor beta 2	rs1042713 G/A	G	Sprint performance of football players; power	[226,227]
*ADRB2*	Adrenoceptor beta 2	rs1042714 C/G	G	Sprint performance of football players; power	[226,227]
*AGT*	Angiotensinogen	rs699 T/C	C	Football player status; sprint performance of football players	[208,226]
*AMPD1*	Adenosine Monophosphate Deaminase 1	rs17602729 C/T	C	Power	[228,229]
*AR*	Androgen Receptor	(CAG)n	≥21	Strength; muscle mass	[230,231]
*BDNF*	Brain-Derived Neurotrophic Factor	rs6265 C/T	C	Coordination; horizontal power, acceleration, and sprint performance in football players	[208,232,233]
*CDKN1A*	Cyclin-Dependent Kinase Inhibitor 1A	rs236448 A/C	C	Power	[234]
*CPNE5*	Copine V	rs3213537 G/A	G	Sprint performance of football players; power	[226,235]
*GALNTL6*	Polypeptide N-acetylgalactosaminyltransferase like 6	rs558129 C/T	T	Power	[236,237]
*HFE*	Homeostatic iron regulator	rs1799945 C/G	G	Endurance	[238]
*IGF2*	Insulin-like growth factor 2	rs680 A/G	G	Sprint performance of football players	[226]
*IGSF3*	Immunoglobulin Superfamily Member 3	rs699785 G/A	A	Power	[239]
*KIBRA*	The kidney and brain expressed protein	rs17070145 C/T	T	Spatial ability; working memory; chess player status	[240,241,242]
*LRPPRC*	Leucine-rich pentatricopeptide repeat cassette	rs10186876 A/G	A	Strength	[243,244,245]
*MCT1*	Monocarboxylate transporter 1	rs1049434 A/T	T (major allele)	Football player status; sprint performance of football players; endurance	[207,246,247,248]
*MMS22L*	Methyl methanesulfonate-sensitivity protein 22-Like	rs9320823 T/C	T	Strength	[244,245,249]
*MYBPC3*	Myosin Binding Protein C3	rs1052373 A/G	G	Endurance	[250]
*NFIA-AS2*	NFIA antisense RNA 2	rs1572312 C/A	C	Endurance	[251,252]
*NOS3*	Nitric oxide synthase 3	rs2070744 T/C	T	Football player status; power	[208,253,254]
*PHACTR1*	Phosphate and actin regulator 1	rs6905419 C/T	C	Strength	[244,245,249]
*PPARA*	Peroxisome proliferator-activated receptor α	rs4253778 G/C	C	Football player status; power; strength	[205,208,255]
*PPARG*	Peroxisome proliferator-activated receptor gamma	rs1801282 C/G	G	Strength	[244,256,257]
*PPARGC1A*	Peroxisome proliferative activated receptor, gamma, coactivator 1 alpha	rs8192678 G/A	G	Endurance	[258,259,260]
*TRHR*	Thyrotropin-releasing hormone receptor	rs7832552 C/T	T	Power	[261,262]
*UCP2*	Uncoupling protein 2	rs660339 C/T	T	Football player status; endurance	[205,263,264]
*VEGFR2*	Vascular endothelial growth factor receptor 2	rs1870377 T/A	A	Endurance	[265,266]

**Table 2:** Assessment type cross-tabulated with testing purpose(s).

	Testing Purpose	Selection/Talent Identification	Monitoring Growth and/or Maturation	Assessing Physiological Fitness	Assessing Sport-Specific Skill	Monitoring Health Status Including Recovery/Fatigue	Quantifying Training Adaptation	Injury Risk Screening
Assessment Type								
Anthropometric								
Stature/mass (e.g., height, weight, limb lengths)		✓	✓					
Body composition (e.g., DXA scanning, skinfolds)			✓			✓	✓	
Physical Capacity								
Aerobic capacity (e.g., Yo-Yo tests, 30-15 Intermittent Fitness Test)		✓		✓		✓	✓	
Muscle strength (e.g., isokinetic dynamometry, adductor squeeze, isometric mid-thigh pull)			✓	✓		✓	✓	✓
Muscle power/reactive strength (e.g., vertical/drop jump tests, single-leg jump/hop tests)			✓	✓		✓	✓	✓
Linear speed (e.g., sprint tests over 10, 20, 30 m)		✓	✓	✓		✓	✓	
Agility (e.g., 505 Test, Illinois Agility Test, Arrowhead Agility Test)		✓	✓	✓	✓			
Repeated sprint ability (e.g., 6–8 repetitions of 25–40 m)		✓	✓	✓		✓	✓	
Biochemical								
Exercise tolerance markers (e.g., blood lactate, lactate threshold)				✓			✓	
Muscle damage markers (e.g., CK, LDH)						✓	✓	✓
Inflammatory markers(e.g., IL-6, TNF-α, CRP)						✓		✓
Immune markers (e.g., WBC count, IgA, cfDNA)						✓		✓
Endocrine markers (e.g., cortisol, testosterone, cortisol/testosterone ratio)						✓		✓
Oxidative markers (e.g., MDA, UA, SOD, TBARS)						✓		✓
Psychological								
Mood state questionnaires (e.g., POMS)						✓		
Recovery questionnaires (e.g., Hooper Index, TQR, RESTQ-Sport)						✓	✓	✓
Return-to-play questionnaires (e.g., RIAI, I-PRRS, ACL-RSI, PRIA-RS)						✓	✓	✓
Injury Risk Factors/Biomechanical								
Movement/balance (e.g., FMS, SIMS, SEBT, YBT)			✓					✓
Landing/cutting mechanics (e.g., LESS, SMAS, CMAS, tuck jump assessment)			✓			✓		✓
Range of motion (e.g., goniometry)							✓	✓
Flexibility (e.g., Thomas Test, Sit-and-Reach Test)							✓	✓
Sport-Specific Skill								
Isolated skill execution (e.g., UGent Dribbling Test, Shuttle Sprint and Dribble Test, LSPT, LSST)		✓				✓	✓	
Simulated skill execution (e.g., small-sides game variations)		✓		✓		✓	✓	
Genetic								
Genetic profiling (e.g., genotyping for single/multiple genetic variants)		✓ *						✓ *

DXA = dual X-ray absorptiometry; CK = creatine kinase; LDH = lactate dehydrogenase; IL-6 = interleukin-6; TNF-α = tumour necrosis factor alpha; CRP = C-reactive protein; WBC = white blood cell; IgA = immunoglobulin A; cfDNA = cell-free DNA; MDA = malondialdehyde; UA = uric acid; SOD = superoxide dismutase; TBARS = thiobarbituric acid reactive substances; POMS = profile of mood states; TQR = total quality of recovery; RESTQ-Sport = Recovery-Stress Questionnaire for Athletes; RIAI = Re-Injury Anxiety Inventory; I-PRRS = Injury Psychological Readiness to Return to Sport; ACL-RSI = Anterior Cruciate Ligament-Return to Sport after Injury; PRIA-RS = Psychological Readiness of Injured Athlete to Return to Sport; FMS = Functional Movement Screen; SIMS = Soccer Injury Movement Screen; SEBT = Star Excursion Balance Test; YBT = Y-Balance Test; LESS = Landing Error Scoring System; SMAS = Sprint Movement Assessment Score; CMAS = Cutting Movement Assessment Score; LSPT = Loughborough Soccer Passing Test; LSST = Loughborough Soccer Shooting Test. * denotes only potential utility for testing purpose(s) due to limited current evidence.

## Data Availability

No data were created during the writing of this manuscript.
